# Decreased 24‐hour Parasympathetic Activity following Intracerebral Hemorrhage: A Key Factor Correlated with Adverse Perihematomal Edema and Poor Functional Outcomes

**DOI:** 10.1002/cns.70727

**Published:** 2026-01-07

**Authors:** Kaijiang Kang, Zeqiang Ji, Yunyi Hao, Jianwei Wu, Yani Zhang, Haiyan Li, Hui Lin, Yuhao Guo, Chuanying Wang, Yang Du, Guangshuo Li, Yongshi Yao, Yijun Lin, Zheng Liu, Jiexin Liu, Xingquan Zhao

**Affiliations:** ^1^ Department of Neurology Beijing Tiantan Hospital, Capital Medical University Beijing China; ^2^ China National Clinical Research Center for Neurological Diseases Beijing China; ^3^ Neurocardiology Clinical and Research Center Beijing Tiantan Hospital, Capital Medical University Beijing China; ^4^ Department of Neurology, Institute of Neuroimmunology Tianjin Medical University General Hospital Tianjin China; ^5^ Department of Clinical Epidemiology and Clinical Trial Capital Medical University Beijing China; ^6^ Department of Internal Medicine MedStar Washington Hospital Center Washington District of Columbia USA; ^7^ Department of Neurology Ulanqab Central Hospital Inner Mongolia China; ^8^ Department of Electrocardiogram Beijing Tiantan Hospital, Capital Medical University Beijing China; ^9^ Department of Medicine Danbury Hospital, Zucker School of Medicine at Hofstra/Northwell Danbury Connecticut USA

**Keywords:** autonomic activity, intracerebral hemorrhage, perihematomal edema

## Abstract

**Aims:**

This study aimed to investigate the association between autonomic activity, assessed by 24‐hour heart rate variability (HRV), and the development of perihematomal edema (PHE) as well as 3‐month functional outcomes following intracerebral hemorrhage (ICH).

**Methods:**

We retrospectively included patients with ICH who underwent 24‐hour electrocardiographic (ECG) monitoring within 14 days of onset from a prospective cohort. HRV parameters were calculated from ECG data. A poor functional outcome at 3 months was defined as a modified Rankin Scale (mRS) score ≥ 3. PHE volume was measured on 7‐day computed tomography scans using 3D Slicer software, and adverse PHE was defined as relative PHE (edema volume/hematoma volume) ≥ 2. Univariate and multivariate logistic regression analyses were performed to identify predictors of adverse PHE and poor outcomes. Partial correlation analysis was conducted to examine the association between HRV parameters and adverse PHE. Five machine‐learning algorithms were applied to develop predictive models for 3‐month poor outcomes.

**Results:**

A total of 312 patients were included, of whom 45.2% (141/312) had poor outcomes at 3 months and 48.6% (122/251) had adverse PHE. Parasympathetic hypoactivity, indicated by low high‐frequency power, was independently associated with poor 3‐month outcomes. In addition, parasympathetic hypoactivity (measured by the root mean square of successive differences between adjacent NN intervals) and relative sympathetic hyperactivity (measured by the ratio of low‐frequency to high‐frequency power) independently predicted adverse PHE. Among the machine‐learning models, the eXtreme Gradient Boosting (XGBoost) algorithm achieved the highest predictive performance for poor 3‐month outcomes, with an AUC of 0.883.

**Conclusions:**

Twenty‐four–hour parasympathetic hypoactivity is associated with adverse PHE and poor functional outcomes following ICH.

## Graphical abstract

This study investigated associations between 24‐hour heart rate variability (HRV)‐assessed autonomic activity and perihematomal edema (PHE) development plus functional outcomes in 312 intracerebral hemorrhage (ICH) patients. Parasympathetic hypoactivity (low high‐frequency power) independently correlated with poor 3‐month outcomes (modified Rankin Scale score ≥ 3). Reduced parasympathetic activity (measured by root mean square of successive differences between adjacent NN intervals) and relative sympathetic hyperactivity (elevated low‐frequency/high‐frequency power ratio) associated with adverse PHE (relative PHE ≥ 2). Among machine‐learning models, eXtreme Gradient Boosting achieved optimal prediction of poor outcomes (AUC = 0.883). Twenty‐four‐hour parasympathetic hypoactivity is associated with adverse PHE and poor functional outcomes post‐ICH.

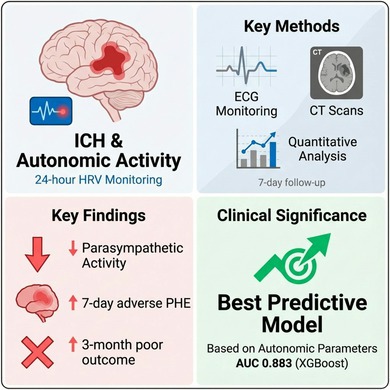

## 1 Introduction

Intracerebral hemorrhage (ICH) is a severe form of stroke, accounting for 10%–30% of all strokes worldwide [1], with a 30‐day mortality rate of 30%–40% [2, 3]. Most survivors experience long‐term disability, placing a substantial burden on families and society [3]. The pathophysiology of ICH involves a cascade of neuroinflammation and secondary brain injury [4]. Perihematomal edema (PHE), a neuroimaging marker of secondary injury, is closely associated with disease progression and poor outcomes, and it has emerged as a key focus of recent research and a potential therapeutic target in ICH [5, 6].

Autonomic dysfunction following ICH is considered an important pathological mechanism contributing to secondary brain injury [7, 8]. This dysfunction, often characterized by sympathetic hyperactivity, has been shown to modulate immune responses and neuroinflammation after ICH, thereby influencing clinical outcomes [9, 10]. Monitoring autonomic activity after ICH and clarifying its relationship with secondary brain injury and long‐term outcomes are therefore of clinical significance. Moreover, regulation therapy of autonomic nervous system (ANS) has been reported to improve functional recovery and outcomes in stroke patients [11, 12]. Stratifying patients according to autonomic activity and correcting the sympathetic–parasympathetic imbalance could potentially improve clinical outcomes [13, 14]. Previous research on autonomic dysfunction in stroke has primarily focused on ischemic stroke and subarachnoid hemorrhage, with relatively few studies addressing ICH [15]. Consequently, the relationship between post‐ICH autonomic activity, secondary injury, and clinical outcomes remains unclear.

Heart rate variability (HRV), defined as the beat‐to‐beat variation in heart rate, is widely recognized as a noninvasive indicator of autonomic activity [16, 17]. Decreased HRV is frequently observed in patients with acute stroke and is associated with increased disability and mortality [18, 19]. Several short‐term electrocardiographic (ECG) monitoring studies have reported an association between reduced HRV and poor outcomes in ICH [20, 21]. However, in these studies, ECG monitoring durations were short, ranging from 10 s to 1 h, which does not adequately capture true HRV across both daytime and nighttime periods [15]. The relationship between 24‐hour HRV and PHE or functional outcomes in patients with ICH remains unclear.

In this study, we aimed to clarify the relationship between 24‐hour autonomic activity and the development of PHE, as well as poor functional outcomes following ICH. Furthermore, we applied machine‐learning algorithms to identify autonomic parameters with significant predictive value and to construct models for predicting poor outcomes after ICH.

## 2 Methods

### 2.1 Study Design and Patient Selection

**FIGURE 1:**
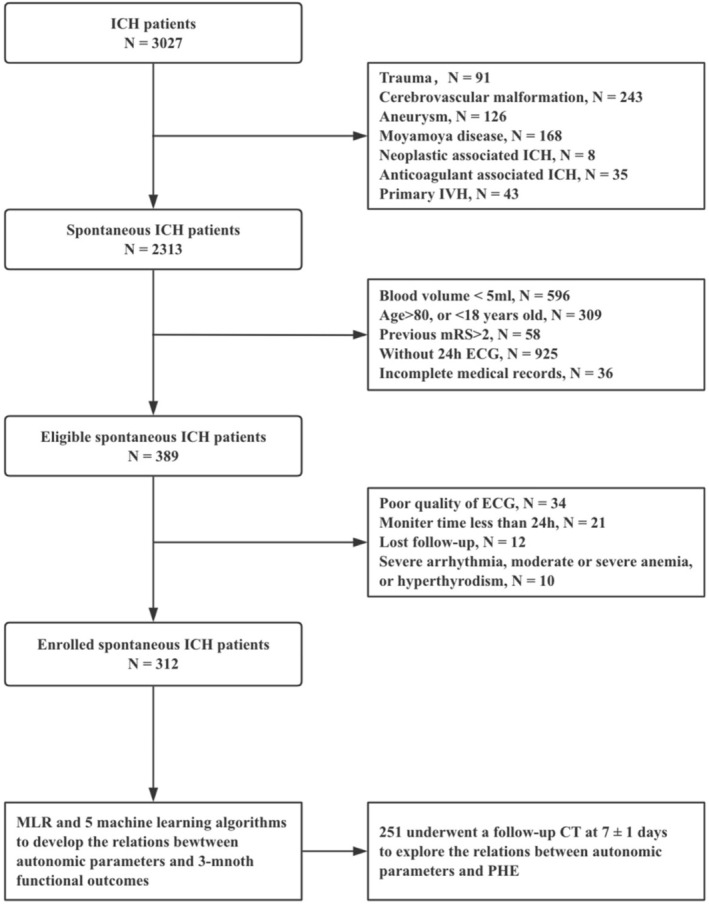
Flowchart of patient selection and study workflow. ECG, electrocardiogram; HRV, heart rate variability; ICH, intracerebral hemorrhage; IVH, intraventricular hemorrhage; MLR, multivariate logistic regression; mRS, modified Rankin Scale; PHE, perihematomal edema.

We retrospectively included patients with ICH who underwent 24‐h ECG monitoring within 14 days of onset, selected from a prospective cohort enrolled between September 2021 and July 2024 at a high‐volume stroke center. The study complied with the principles of the Declaration of Helsinki and the World Medical Association and was approved by the Institutional Review Board of Beijing Tiantan Hospital (KY2021‐025‐01). The inclusion criteria were: (1) age 18–80 years; (2) spontaneous ICH confirmed by non‐contrast computed tomography (NCCT); (3) initial CT performed within 72 h of symptom onset; (4) baseline hematoma volume ≥ 5 mL; (5) pre‐ICH modified Rankin Scale (mRS) score ≤ 2; and (6) informed consent obtained from participants or their legal representatives. The exclusion criteria were: (1) secondary ICH due to trauma, cerebrovascular malformation, intracranial aneurysm, moyamoya disease, neoplastic lesions, or other causes; (2) antithrombotic‐related ICH or coagulopathies; (3) primary intraventricular hemorrhage; (4) hyperthyroidism, moderate or severe anemia, or previously diagnosed or Holter‐detected severe arrhythmias (including atrial fibrillation, ventricular fibrillation, frequent premature beats, and second or third‐degree atrioventricular block); (5) absence of 24‐hour ECG records within 14 days of onset; and (6) missing essential clinical or radiological data. The patient selection flow chart is shown in Figure 1.

### 2.2 Clinical Characteristics, Follow‐Up, and Outcomes

Clinical data were recorded in the prospective cohort database and included demographic characteristics, medical history, baseline GCS and NIHSS scores, time from onset to CT and ECG, and hematologic test results. The ICH score was evaluated according to the published definition [22]. All patients were followed up face‐to‐face or by telephone 3 months after ICH onset, and a modified Rankin Scale (mRS) score ≥ 3 was defined as a poor outcome.

### 2.3 Radiological Information Acquisition and Interpretation

Baseline and follow‐up NCCT scans (16‐slice CT scanner, Brilliance iCT, Philips Healthcare, Cleveland, OH) were performed using the following parameters: 120 kVp, 310 mAs, 5 mm slice thickness, and image size of 512 × 512 pixels. Hematoma location and the presence of intraventricular hemorrhage were documented. Follow‐up CT was conducted at 7 ± 1 days after ICH onset.

**FIGURE 2:**
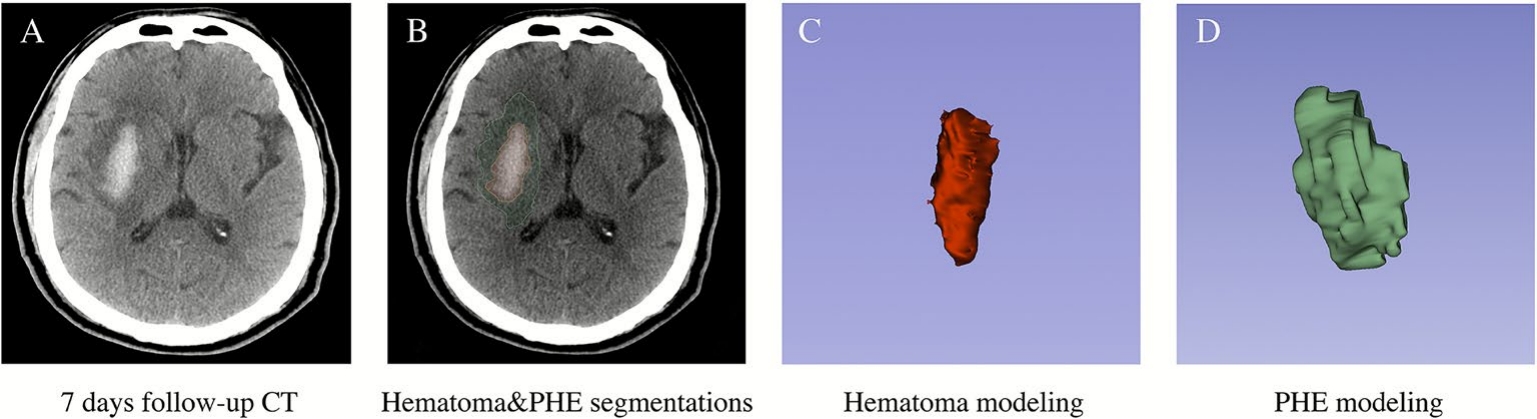
Segmentation and modeling of hematoma and perihematomal edema using 3D Slicer. Panels A–D are images from a follow‐up CT scan of a patient with ICH. Panels A and B show hematoma segmentation and modeling performed with 3D Slicer software on a 7‐day follow‐up CT. Panel C shows hematoma modeling and volume calculations, and Panel D shows PHE modeling and volume calculations. CT, computed tomography; ICH, intracerebral hemorrhage; PHE, perihematomal edema.

Baseline and follow‐up hematoma and PHE volumes were measured using 3D Slicer software (version 5.6.2, www.slicer.org, Harvard University, USA), which semi‐automatically delineated their boundaries with a level tracing tool (Figure 2). PHE was defined as a hypodense region adjacent to the hematoma [23]. In each slice, the software semi‐automatically identified pixels, with CT values of 5–33 HU defined as edema [24] and 44–100 HU defined as hematoma [25]. After manual boundary adjustment in 3D Slicer, 3D models of the hematoma and PHE were generated, and their volumes were automatically calculated.

Two experienced neurologists, blinded to the clinical outcomes and HRV parameters, independently performed the measurements. The average of their results was used for statistical analysis. Detailed methodology has been reported in our previous publication [26]. Intra‐class correlation coefficients (ICCs) were calculated to assess measurement reliability, with ICC > 0.80 (range 0–1) indicating excellent agreement. Relative PHE (rPHE) was defined as perihematomal edema volume divided by hematoma volume on the 7‐day CT [6]. The optimal cut‐off value of rPHE for predicting poor outcomes was determined by receiver operating characteristic (ROC) curve analysis. The Youden index (sensitivity + specificity −1) was used to identify the cut‐off point that maximized the sum of sensitivity and specificity. The adverse PHE was defined based on this cut‐off value.

### 2.4 ECG Data Acquisition and Analysis

All enrolled patients underwent 24‐hour ECG monitoring using a 3‐channel, 5‐lead digital Holter recorder (BI‐9800, Biomedical Inc., Shenzhen, China). Monitoring began before 8:00 AM on the first day and ended after 8:00 AM on the second day to ensure continuous 24‐hour coverage. All recordings were obtained while patients remained in a bedridden state in either the stroke unit or the neurological intensive care unit.

Continuous ECG data were stored and analyzed using an ECG workstation provided by the Holter recorder manufacturer (Biomedical Inc., Shenzhen, China). The system automatically identified normal QRS complexes and abnormal waveforms, followed by manual review to confirm and exclude abnormal segments, retaining only NN intervals where both adjacent beats were typical QRS complexes. Additionally, differences between adjacent NN intervals were limited to ≤ 20%. After this process, HRV parameters were calculated using the NN interval sequences.

Time‐domain parameters were automatically calculated according to standard definitions: mean NN interval, standard deviation of all NN intervals (SDNN), root mean square of successive differences between adjacent NN intervals (RMSSD), number of adjacent NN intervals differing by more than 50 ms (NN50), and percentage of adjacent NN intervals differing by more than 50 ms (pNN50) [27].

Frequency‐domain parameters were calculated using the Fast Fourier Transform (FFT) algorithm. The procedure involved resampling NN interval data at 4 Hz, performing FFT on the resampled data to obtain the power spectrum, and calculating power values for high‐frequency (HF), low‐frequency (LF), very low‐frequency (VLF), ultra‐low‐frequency (ULF), total power (TP), and the LF/HF ratio, according to specified frequency bands [27].

### 2.5 Statistical Analysis

Continuous variables were expressed as median (interquartile range [IQR]) or mean ± standard deviation and compared using the Mann–Whitney *U* test or Student's *t*‐test, as appropriate. Categorical variables were expressed as count (percentage) and compared using the *χ* ^2^ test or Fisher's exact test. Variables with *p* < 0.05 in the univariate analysis comparing patients with good versus poor outcomes, or those with versus without adverse edema, were entered into multivariate logistic regression analysis. To assess the association between rPHE and autonomic parameters in patients with ICH, partial correlation analyses were performed with adjustment for age, sex, baseline hematoma volume,NIHSS GCS score, and time from onset to ECG. A two‐tailed *p* < 0.05 was considered statistically significant. All statistical analyses were performed using RStudio software (https://www.r‐project.org/, version 4.2.3).

### 2.6 Machine‐Learning Algorithms and Prediction Model Construction

After data preprocessing, the cohort was randomly split into training and testing sets in a 7:3 ratio. Five machine‐learning algorithms were applied for model development: logistic regression (LR), support vector machine (SVM), random forest (RF), Adaptive Boosting (AdaBoost), and eXtreme Gradient Boosting (XGBoost). Bayesian optimization was used for hyperparameter tuning, and model performance was evaluated using 5‐fold cross‐validation. Evaluation metrics included the area under the receiver operating characteristic curve (AUC), average precision (AP), accuracy, precision, recall, and F1‐score to determine the optimal model. DeLong's test was used to compare the performance of the five machine‐learning algorithms with the ICH score. SHapley Additive exPlanations (SHAP) were used to assess feature importance and improve model interpretability.

The top five variables ranked by importance in each of the five algorithms were summarized to highlight the predictive significance of autonomic parameters for functional outcomes. All modeling processes were conducted using RStudio software (https://www.r‐project.org/, version 4.2.3).

## 3 Results

### 3.1 Baseline Characteristics of Patients

A total of 312 patients, with a median age of 57.0 years (IQR, 49–65.3), were enrolled between September 2021 and July 2024, including 238 (76.3%) males and 74 (23.7%) females. The flowchart of the patient selection process is shown in Figure 1. The median baseline GCS and NIHSS scores were 15 (IQR, 15–15) and 5 (IQR, 2–10), respectively. The median times from ICH onset to hospital admission and from ICH onset to 24‐h ECG were 6.6 h (IQR, 4.0–12.8) and 6.0 days (IQR, 5.0–7.0), respectively. The median baseline hematoma volume was 17.5 mL (IQR, 9.7–31.6), and 90 patients (28.8%) had intraventricular hemorrhage (IVH). All baseline characteristics are summarized in Table S1.

### 3.2 Clinical Outcomes and Associated Factors

Among the enrolled patients, 141 (45.2%) had poor outcomes at 3 months. Compared with those with good outcomes, patients with poor outcomes had a higher baseline NIHSS score (10 vs. 3, *p* < 0.001), lower baseline GCS score (14 vs. 15, *p* < 0.001), larger baseline hematoma volume (32.7 mL vs. 17.3 mL, *p* < 0.001), and greater 7‐day rPHE (2.7 vs. 1.7, *p* < 0.001).

Parasympathetic hypoactivity, as reflected by decreased 24‐hour HRV parameters, was associated with poor outcomes. In the time‐domain analysis, patients with poor outcomes had lower RMSSD (20.0 ms vs. 23.0 ms, *p* = 0.001), NN50 (2020.0 vs. 2779.0, *p* = 0.026), and pNN50 (2.4% vs. 3.3%, *p* = 0.004) values. In the frequency‐domain analysis, poor outcome was associated with lower HF (87.6 ms^2^ vs. 108.5 ms^2^, *p* = 0.006) and a higher LF/HF ratio (2.5 vs. 2.0, *p* = 0.037). No significant differences were found in other clinical, radiological, or autonomic characteristics (Table 1).

**TABLE 1:** Multivariate logistic regression results for 3‐month poor outcomes.

	*p*	OR	95% CI
NIHSS	< 0.001*	1.243	1.158–1.336
Baseline hematoma volume	< 0.001*	1.109	1.077–1.142
24 h HF	0.017*	0.998	0.996–0.999
GCS	0.545	1.083	0.836–1.403
24 h LF/HF	0.231	1.108	0.937–1.310
24 h RMSSD	0.499	0.984	0.939–1.031
24 h pNN50	0.302	1.023	0.980–1.068
Onset to ECG time	0.189	1.112	0.945–1.333

*Note:* NIHSS, baseline hematoma volume, and 24 h HF remained significant after adjustment for variables with *p* < 0.05 in univariate analysis (including baseline volume, GCS, NIHSS, 24 h RMSSD, 24 h pNN50, 24 h HF, 24 h LF/HF), and onset to ECG time. * for statistical significance.

Abbreviations: CI, confidence interval; GCS, Glasgow Coma Scale; HF, high‐frequency; LF, low‐frequency; NIHSS, National Institutes of Health Stroke Scale; NN50, number of adjacent NN intervals differing by more than 50 ms; OR, odds ratio; pNN50, percentage of adjacent NN intervals differing by more than 50 ms; RMSSD, root mean square of successive differences between adjacent NN intervals.

Multivariate LR analysis showed that HF (OR, 0.998; 95% CI, 0.996–0.999; *p* = 0.017), NIHSS score (OR, 1.243; 95% CI, 1.158–1.336; *p* = 0.009), and baseline hematoma volume (OR, 1.109; 95% CI, 1.077–1.142; *p* < 0.001) were independently associated with poor 3‐month outcomes after adjusting for variables with *p* < 0.05 in the univariate analysis (including GCS score, 24‐h RMSSD, 24‐h pNN50, and 24‐h LF/HF). Because NN50 and pNN50 are highly correlated in calculation principles and methods, only one was included in the multivariate analysis, and the same results were obtained regardless of which was used (Table 1). * for statistical significance.

### 3.3 PHE and Associated Factors

Of the 312 patients, 251 (80.4%) underwent CT scans at 7 ± 1 days after onset. The median absolute and relative PHE volume were 20.2 mL (IQR, 5.3–18.0) and 2.0 (IQR, 1.6–2.8), respectively. A rPHE of ≥ 2 was defined as adverse edema, based on the cut‐off value (2.016) differentiating poor outcomes in this cohort. Among the 251 patients, 122 had adverse PHE. These patients demonstrated larger baseline hematoma volumes, higher NIHSS scores, and lower GCS scores (*p* < 0.05). They also exhibited reduced parasympathetic activity, as reflected by lower RMSSD (19.0 ms vs. 25.0 ms, *p* < 0.001), lower HF (87.0 ms^2^ vs. 137.5 ms^2^, *p* < 0.001), and higher LF/HF ratios (2.8 vs. 1.8, *p* < 0.001; Table 1). In multivariate analysis, RMSSD (OR, 0.960; 95% CI, 0.933–0.988; *p* = 0.005), LF/HF (OR, 1.428; 95% CI, 1.196–1.705; *p* < 0.001), NIHSS score (OR, 1.169; 95% CI, 1.091–1.251; *p* < 0.001), deep hematoma (OR, 2.332; 95% CI, 1.094–4.971; *p* = 0.028), and onset to ECG time (OR, 1.305; 95% CI, 1.085–1.570; *p* = 0.005) remained independently associated with adverse PHE (Table 2).

**TABLE 2:** Multivariate logistic regression results for adverse PHE.

	*p*	OR	95% CI
NIHSS	< 0.001*	1.169	1.091–1.251
24 h RMSSD	0.005	0.960	0.933–0.988
24 h LF/HF	< 0.001*	1.428	1.196–1.705
Deep hematoma	0.028	2.332	1.094–4.971
Onset to ECG time	0.005	1.305	1.085–1.570
Baseline hematoma volume	0.454	1.008	0.987–1.030
24 h HF	0.331	0.999	0.996–1.001
GCS	0.786	1.033	0.818–1.304

*Note:* NIHSS, 24 h RMSSD, and 24 h LF/HF remained significant after adjustment for variables with *p* < 0.05 in the univariate analysis and onset to ECG time.* for statistical significance.

Abbreviations: CI, confidence interval; GCS, Glasgow Coma Scale; HF, high‐frequency; IVH, intraventricular hemorrhage; LF, low‐frequency; NIHSS, National Institutes of Health Stroke Scale; NN50, number of adjacent NN intervals differing by more than 50 ms; OR, odds ratio; pNN50, percentage of adjacent NN intervals differing by more than 50 ms; RMSSD, root mean square of successive differences between adjacent NN intervals; TP, total power.

### 3.4 Partial Correlation Analysis Between Autonomic Parameters and rPHE

**FIGURE 3:**
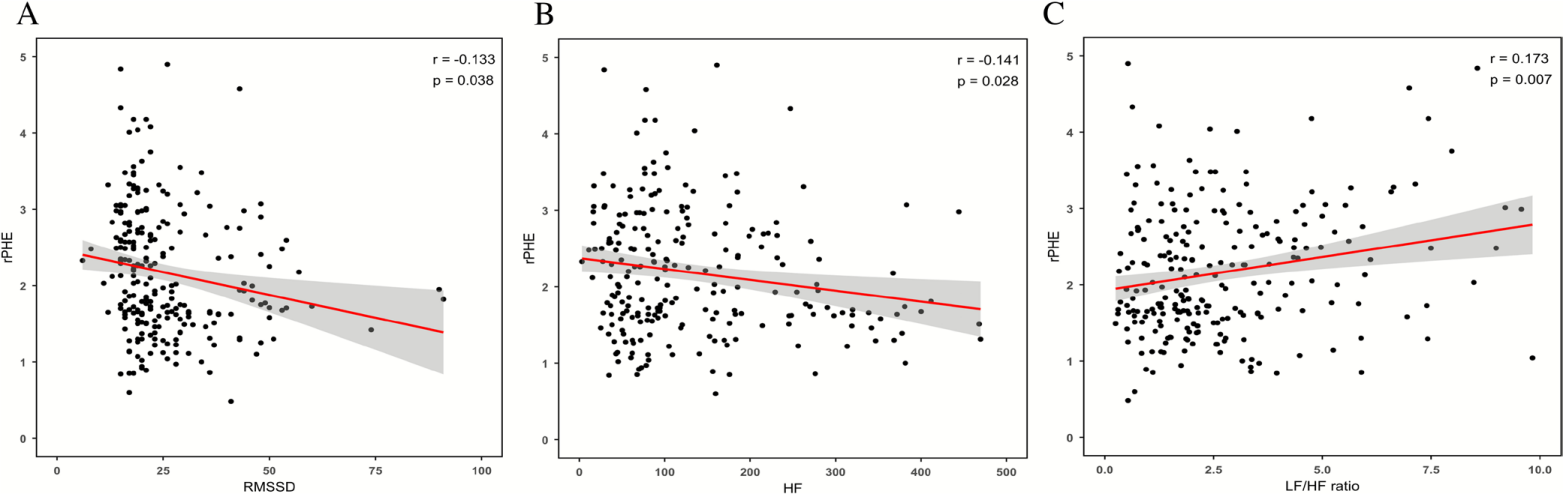
Partial correlations between autonomic parameters and rPHE. In patients with ICH, lower parasympathetic activity was associated with higher relative PHE. Specifically: (A) higher rPHE was correlated with lower RMSSD (r = −0.133; *p* = 0.038), (B) lower HF (r = −0.141; *p* = 0.028), and (C) higher LF/HF (*r* = 0.173; *p* = 0.007) after adjustment for age, sex, baseline hematoma volume, NIHSS, GCS, and time from onset to ECG. The solid line represents the linear regression, and the shaded area represents the 95% confidence interval. HF, high‐frequency; ICH, intracerebral hemorrhage; LF, low‐frequency; RMSSD, root mean square of successive differences between adjacent NN intervals; rPHE, relative perihematomal edema.

Parasympathetic hypoactivity was found to be associated with higher rPHE. Specifically, higher rPHE correlated with lower RMSSD (*r* = −0.133; *p* = 0.038), lower HF (*r* = −0.141; *p* = 0.028), and higher LF/HF (*r* = 0.173; *p* = 0.007), after adjusting for age, sex, baseline hematoma volume, NIHSS, GCS, and the time from onset to ECG. No significant correlations were observed between rPHE and other autonomic parameters (Figure 3).

### 3.5 Machine‐Learning Algorithms for Predicting Poor Outcomes

**FIGURE 4:**
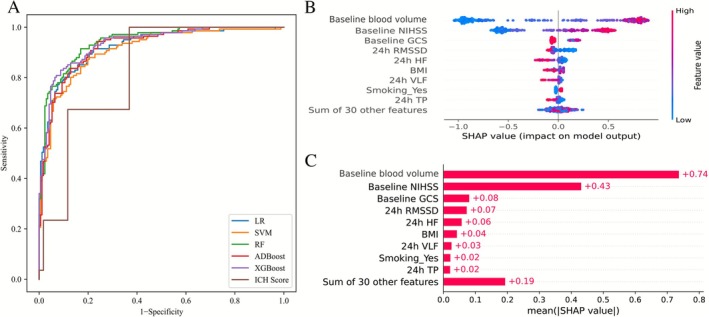
Comparison of five machine‐learning models with the ICH score. Five machine‐learning models were developed: Logistic regression (LR), support vector machine (SVM), random forest (RF), Adaptive Boosting (AdaBoost), and eXtreme Gradient Boosting (XGBoost). These models were compared with the ICH score (A). Variables included in the XGBoost model are shown in panel B, and SHAP values illustrating feature importance in the XGBoost model are shown in panel C. BMI, body mass index; GCS, Glasgow Coma Scale; HF, high‐frequency; ICH, intracerebral hemorrhage; NIHSS, National Institutes of Health Stroke Scale; RMSSD, root mean square of successive differences between adjacent NN intervals; SHAP, SHapley Additive exPlanations; TP, total power; VLF, very low‐frequency.

After data preprocessing, the cohort was randomly split into training and testing sets in a 7:3 ratio. All five machine‐learning algorithms demonstrated robust performance after Bayesian optimization outperformed the ICH score (AUC = 0.656, 95% CI: 0.595–0.718; Figure 4A; Tables S2 and S3).

Among the five models, XGBoost achieved the best performance, with an AUC of 0.883 (95% CI: 0.833–0.934) and an AP of 0.867 (95% CI: 0.813–0.921). Notably, HF and RMSSD emerged as the most influential autonomic parameters within XGBoost, both showing a negative correlation with poor outcomes (Figure 4B,C). Moreover, HF and RMSSD ranked among the top five most important variables in three of the five algorithms (Table 3, Figure S1).

**TABLE 3:** Top five variable importance rankings in five machine‐learning algorithms.

Variable	LR	SVM	RF	AdaBoost	XGBoost	Sum
24 h SDNN	√					1
24 h RMSSD			√	√	√	3
24 h NN50						0
24 h pNN50						0
Triangular index						0
24 h ULF						0
24 h VLF	√	√		√		3
24 h LF						0
24 h HF			√	√	√	3
24 h TP		√				1
LF/HF	√	√				2

Abbreviations: AdaBoost, adaptive boosting; AUC, area under the curve; HF, high‐frequency; ICH, intracerebral hemorrhage; LF, low‐frequency; LR, Logistic Regression; NN50, number of adjacent NN intervals differing by more than 50 ms; pNN50, percentage of adjacent NN intervals differing by more than 50 ms; RF, random forest; RMSSD, root mean square of successive differences between adjacent NN intervals; SDNN, standard deviation of NN intervals; SVM, support vector machine; TP, total power; ULF, ultralow‐frequency; VLF, very low‐frequency; XGBoost, eXtreme Gradient Boosting.

## 4 Discussion

To the best of our knowledge, this is the first and largest cohort study to conduct long‐term (24‐hour) monitoring of autonomic nervous activity in patients with intracerebral hemorrhage. We found that autonomic parameters reflecting parasympathetic hypoactivity (including RMSSD and HF) were associated with 7‐day adverse edema and poor 3‐month outcomes following ICH. In addition, sympathetic–parasympathetic imbalance, indicated by a higher LF/HF ratio, was also associated with adverse edema.

Post‐stroke autonomic dysfunction has been linked to neurological functional outcomes and a range of complications [28, 29], including cerebro‐cardiac syndrome [30], blood pressure fluctuations [31], hyperglycemia [32], thermoregulatory dysfunction [21], immunosuppression and infection, and digestive dysfunction [7]. The underlying mechanism is thought to involve disruption of the central autonomic network (CAN), which integrates viscerosensory and humoral inputs and coordinates sympathetic and parasympathetic activity to regulate physiological and metabolic functions under normal conditions [8]. With both primary and secondary injuries affecting the CAN, ICH can trigger autonomic dysfunction or sympathetic–parasympathetic imbalance [33]. Autonomic dysregulation has been identified as a key pathway driving secondary inflammation after stroke. Typically, post‐stroke sympathetic hyperactivity mobilizes immune cells from peripheral reservoirs such as the spleen or promotes their generation in bone marrow, increasing circulating immune cells [9, 34]. In contrast, anti‐inflammatory effects mediated by acetylcholine in cholinergic pathways have recently been described: acetylcholine rapidly inhibits macrophages and reduces pro‐inflammatory cytokine secretion [35, 36]. Therefore, sympathetic hyperactivity and parasympathetic hypoactivity may contribute to an ANS‐mediated pro‐inflammatory response following ICH, thereby promoting adverse PHE and ultimately leading to poor functional outcomes.

Several techniques have been developed to quantitatively evaluate autonomic nervous activity, including HRV, baroreflex sensitivity (BRS) [31], skin sympathetic nerve activity (SSNA) [37], sympathetic skin response (SSR) [38], and plasma catecholamine concentration [39]. Among these methods, HRV has gained increasing attention for detecting autonomic dysfunction because of its accessibility and availability across a wide range of monitoring devices [40]. HRV reflects the beat‐to‐beat variation in cardiac intervals and can be analyzed using time‐domain, frequency‐domain, and nonlinear methods. Most studies have demonstrated that reduced HRV, whether in time‐domain, frequency‐domain, or nonlinear parameters [41], is independently associated with poor outcomes in stroke patients [18, 19, 21]. However, research has primarily focused on ischemic stroke [18] and SAH [29], with relatively few studies examining ICH. Although decreased HRV has been linked to poor outcomes after ICH [20, 21, 37, 42], these studies generally used short monitoring durations (10 min to 1 h [17]), which are insufficient to capture full daily variability. Because autonomic activity follows circadian rhythm, 24‐h HRV provides a more comprehensive and objective measure of autonomic function, encompassing both diurnal and nocturnal fluctuations and reducing bias from monitoring time selection [40]. Some studies have used pulse rate variability as a substitute for HRV; however, pulse does not equal heartbeat, as it is influenced by vascular conditions that affect pulse transmission. In the present study, decreased 24‐h HF, identified in both multivariate LR and multiple machine‐learning algorithms as a marker of parasympathetic hypoactivity across the full day, emerged as a strong predictor of poor outcomes after ICH.

In contrast, a post hoc analysis of the ATACH‐2 trial reported that increased heart rate average real variability (HR‐ARV) within 24 h after ICH was independently associated with poor outcomes. However, this conclusion was based on heart rate values recorded every 15 min rather than analysis of all NN intervals using specific algorithms, which do not strictly represent HRV and therefore does not contradict our findings [43].

RMSSD, the root mean square of successive differences between adjacent NN intervals, reflects short‐term and rapid heart rate fluctuations and is primarily mediated by vagal (parasympathetic) activity [44]. Decreased RMSSD has been identified as a predictor of poor functional outcomes. In our cohort, we further examined correlations between parasympathetic parameters and rPHE and, for the first time, demonstrated that parasympathetic hypoactivity indicated by 24‐h RMSSD was strongly associated with adverse PHE.

As a neuroimaging marker of secondary injury, PHE has been recognized as an important therapeutic target for improving ICH outcomes [6]. The findings of this study suggest that autonomic dysfunction after ICH contributes to PHE, which in turn is linked to poor outcomes. Therefore, correcting autonomic dysfunction after ICH, such as through vagal nerve stimulation or sympathetic inhibition, represents a potential novel treatment strategy [45]. The current comprehensive ICH management protocols, the Code ICH, recommended for the acute phase of ICH have been shown to mitigate secondary brain injury and long‐term prognosis [46, 47]. Autonomic assessments during the acute phase of ICH and targeting interventions on parasympathetic hypoactivity may have the potential to improve PHE and long‐term outcomes, which represents a novel improvement to existing management strategies.

In this study, five machine‐learning algorithms were used to identify HRV parameters most strongly associated with poor outcomes. All five models significantly outperformed the traditional ICH score in predicting outcomes. Although autonomic parameters are less widely available than conventional clinical variables and rarely used for outcome prediction after ICH, our use of advanced machine‐learning analytics highlights their prognostic value and supports the overall findings of this study.

This study has some limitations. First, the sample size was relatively small, and all patients were recruited from a single center, which may introduce selection bias. Second, even though the majority of patients (87.8%) underwent ECG between 4 and 8 days post‐ICH, the time from onset to ECG ranged from 2 to 13 days, which may confound the association as autonomic function and PHE dynamics vary over time, in spite of the adjustment. Third, further research is needed to evaluate HRV changes among different time periods.

## 5 Conclusions

The 24‐hour parasympathetic hypoactivity is associated with adverse PHE and poor functional outcomes following ICH. Modulation of the ANS represents a promising potential therapeutic strategy for ICH.

## Author Contributions

All authors made a significant contribution to the work reported, whether that is in the conception, study design, execution, acquisition of data, analysis and interpretation, or in all these areas; took part in drafting, revising, or critically reviewing the article; gave final approval of the version to be published; have agreed on the journal to which the article has been submitted; and agree to be accountable for all aspects of the work. All authors have read and approved the final submitted manuscript.

## Funding

This work was supported by the National Natural Science Foundation of China (Grant number: 82471489 to K.J.K., Grant number: 82371302 to X.Q.Z., Grant number: 82401452 to G.S.L.), Health China·BuChang ZhiYuan Public Welfare Projects for Heart and Brain Health (HIGHER 2023074 to K.J.K.), Noncommunicable Chronic Disease‐National Science and Technology Major Project (2023ZD0505405 to J.W.W.).

## Ethics Statement

The study complied with the principles of the Declaration of Helsinki and the World Medical Association and was approved by the Institutional Review Board of Beijing Tiantan Hospital (KY2021‐025‐01).

## Consent

All Consent Statements have been acquired.

## Conflicts of Interest

The authors declare no conflicts of interest.

## Supporting information


**Table S1:** Baseline variables of patients with good or poor 3‐month outcomes and adverse or non‐adverse perihematomal edema (PHE).
**Table S2:** Model performance for five machine learning algorithms and comparison with ICH score.
**Table S3:** Optimal hyperparameters for five machine learning algorithms.
**Figure S1:** Other 4 machine learning models.

## Data Availability

The data of this study will be available on reasonable request from the corresponding author.
