# Supplementary material for: Decreased 24‐hour Parasympathetic Activity following Intracerebral Hemorrhage: A Key Factor Correlated with Adverse Perihematomal Edema and Poor Functional Outcomes
Source: CNS Neurosci Ther. 2026 Jan 7;32(1):e70727. doi: 10.1002/cns.70727 (PMC12778931; doi:10.1002/cns.70727)
Supplement: Supplementary file 1 — Table S1: Baseline variables of patients with good or poor 3‐month outcomes and adverse or non‐adverse perihematomal edema (PHE). Table S2: Model performance for five machine learning algorithms and comparison with ICH score. Table S3: Optimal hyperparameters for five machine learning algorithms. Figure S1: Other 4 machine learning models. [file CNS-32-e70727-s001.docx]

**Supplementary Table 1. Baseline variables of patients with good or poor 3-month outcomes and adverse or non-adverse perihematomal edema (PHE).**

|  | Total cohort  N=312 | Good outcome  N=171 | Poor outcome  N=141 | *p* | Non-adverse PHE  N=129 | Adverse PHE  N=122 | *p* |
| --- | --- | --- | --- | --- | --- | --- | --- |
| Age, years | 57.0 (49.0, 65.3) | 57.0 (48.0, 65.0) | 58.0 (50.0, 65.0) | 0.404 | 56.0 (49.0,65.5) | 58.0 (50.8,65.3) | 0.616 |
| Male, n (%) | 238 (76.3) | 128 (74.9) | 110 (78.0) | 0.603 | 102 (79.1) | 90 (73.8) | 0.322 |
| BMI, kg/m^2^ | 22.9 (20.1, 25.9) | 22.5 (19.6,26.4) | 23.1 (20.6,25.6) | 0.731 | 22.9 (20.1,26.2) | 23.4 (20.4,26.1) | 0.553 |
| Hypertension, n (%) | 218 (69.9) | 123 (71.9) | 95 (67.4) | 0.454 | 89 (69.0) | 82 (67.2) | 0.762 |
| Diabetes mellitus, n (%) | 59 (18.9) | 35 (20.5) | 24 (17.0) | 0.53 | 26 (20.2) | 22 (18.0) | 0.669 |
| CHD, n (%) | 19 (6.1) | 13 (7.6) | 6 (4.3) | 0.321 | 6 (4.7) | 6 (4.9) | 0.921 |
| Ischemic stroke, n (%) | 50 (16.0) | 24 (14.0) | 26 (18.4) | 0.368 | 19 (14.7) | 19 (15.6) | 0.852 |
| Smoking, n (%) | 112 (35.9) | 54 (31.6) | 58 (41.1) | 0.103 | 41 (31.8) | 49 (40.2) | 0.166 |
| Alcohol consumption, n (%) | 114 (36.5) | 61 (35.7) | 53 (37.6) | 0.817 | 48 (37.2) | 43 (35.3) | 0.746 |
| α-blocker use, n (%) | 12 (3.8) | 4 (2.3) | 8 (5.7) | 0.219 | 4 (3.1) | 6 (4.9) | 0.462 |
| β-blocker use, n (%) | 32 (10.3) | 16 (9.4) | 16 (11.3) | 0.697 | 10 (7.8) | 8 (6.6) | 0.714 |
| ACEI use, n (%) | 7 (2.2) | 5 (2.9) | 2 (1.4) | 0.61 | 3 (2.3) | 3 (2.5) | 0.945 |
| ARB use, n (%) | 171 (54.8) | 89 (52.0) | 82 (58.2) | 0.335 | 72 (55.8) | 66 (54.1) | 0.785 |
| CCB use, n (%) | 211 (67.6) | 110 (64.3) | 101 (71.6) | 0.17 | 81 (62.8) | 88 (72.1) | 0.115 |
| Diuretics use, n (%) | 92 (29.5) | 50 (29.2) | 42 (29.8) | 1 | 40 (31.0) | 37 (30.3) | 0.907 |
| Sacubitril Valsartan, n (%) | 17 (5.4) | 8 (4.7) | 9 (6.4) | 0.682 | 8 (6.2) | 6 (4.9) | 0.658 |
| Onset to CT, h | 6.6 (4.0, 12.8) | 7.0 (4.0, 12.4) | 6.5 (4.0, 13.0) | 0.664 | 7.0 (4.0,12.5) | 6.8 (4.0,12.2) | 0.644 |
| Baseline GCS | 15.0 (14.8, 15.0) | 15.0 (15.0, 15.0) | 14.0 (14.0, 15.0) | <0.001* | 15.0 (15.0,15.0) | 15.0 (14.0,15.0) | 0.001* |
| Baseline NIHSS | 5.0 (2.0, 10.0) | 3.0 (2.0, 6.0) | 10.0 (6.0, 12.0) | <0.001* | 4.0 (2.0,8.0) | 9.0 (3.0,12.0) | <0.001* |
| Baseline SBP, mmHg | 164.0 (148.0, 187.3) | 163.0 (144.5, 186.5) | 166.0 (149.0, 189.0) | 0.426 | 162.0 (144.0,184.5) | 165.0 (149.0,190.5) | 0.475 |
| Baseline DBP, mmHg | 95.0 (83.0, 103.0) | 95.0 (83.0, 103.0) | 95.0 (83.0, 104.0) | 0.82 | 94.0 (82.5,102.0) | 94.0 (80.0,105.0) | 0.434 |
| Baseline HV, mL | 17.5 (9.7, 31.6) | 11.2 (7.4, 17.0) | 30.4 (20.5, 39.4) | <0.001* | 13.0 (8.4,23.3) | 22.9 (13.8,34.5) | <0.001* |
| IVH, n (%) | 90 (28.8) | 43 (25.1) | 47 (33.3) | 0.143 | 38 (29.46) | 35 (28.7) | 0.893 |
| Hematoma position, n (%) | | | | 0.131 |  |  | <0.001* |
| deep hematoma | 222 (71.2) | 120 (70.2) | 102 (72.3) |  | 89 (69.0) | 90 (73.9) |  |
| lobar hematoma | 68 (21.8) | 34 (19.9) | 34 (24.1) |  | 23 (17.8) | 32 (26.2) |  |
| cerebellar hematoma | 20 (6.4) | 15 (8.8) | 5 (3.5) |  | 16 (12.4) | 0 (0.0) |  |
| brainstem hematoma | 2 (0.6) | 2 (1.2) | 0 (0.0) |  | 1 (0.8) | 0 (0.0) |  |
| Onset to ECG, days | 6.0 (5.0, 7.0) | 6.0 (5.0, 7.0) | 6.0 (4.0, 7.0) | 0.927 | 6.0 (5.0,7.0) | 6.0 (5.0,7.0) | 0.078 |
| Mean heart rate | 82.0 (75.8, 90.3) | 82.0 (75.5, 90.5) | 82.0 (76.0, 90.0) | 0.765 | 82.0 (75.0,89.5) | 83.0 (76.0,92.0) | 0.423 |
| 24h SDNN, ms | 83.0 (66.8, 105.3) | 86.0 (68.0, 108.50) | 80.0 (66.0, 97.0) | 0.096 | 83.0 (68.0,106.0) | 82.5 (66.0,101.3) | 0.443 |
| 24h RMSSD, ms | 22.0 (18.0, 30.0) | 23.0 (19.0, 31.0) | 20.0 (17.0, 28.0) | 0.001* | 25.0 (20.0,33.0) | 19.0 (16.0,23.3) | <0.001* |
| 24h NN50 | 2321.5 (781.0, 7210.8) | 2779.0 (1022.5, 8011.5) | 2020.0 (574.0, 6623.0) | 0.026* | 2315.0 (930.0,7642.0) | 2126.5 (561.8,7210.8) | 0.174 |
| 24h pNN50, % | 2.9 (1.1, 10.3) | 3.3 (1.2, 11.0) | 2.4 (0.8, 8.6) | 0.042* | 2.8 (1.2,10.5) | 2.6 (0.8,9.9) | 0.291 |
| 24h Triangular index | 13.7 (10.6, 18.0) | 14.2 (10.6, 18.4) | 13.1 (10.8, 17.4) | 0.079 | 13.9 (10.6,18.0) | 14.0 (10.9,18.4) | 0.884 |
| 24h ULF, ms^2^ | 19.6 (12.6, 29.2) | 21.6 (14.2, 30.2) | 19.0 (11.0, 28.0) | 0.075 | 19.6 (13.9,31.1) | 19.5 (10.8,26.8) | 0.143 |
| 24h VLF, ms^2^ | 849.4 (509.5, 1336.5) | 893.5 (518.8, 1344.2) | 766.0 (454.9, 1333.6) | 0.312 | 855.9 (505.0,1369.9) | 781.9 (462.2,1238.8) | 0.339 |
| 24h LF, ms^2^ | 239.2 (132.0, 441.2) | 243.7 (147.4, 482.5) | 224.0 (117.1, 401.3) | 0.133 | 235.0 (128.5,456.5) | 226.6 (138.5,401.8) | 0.655 |
| 24h HF, ms^2^ | 101.7 (60.2, 202.9) | 108.5 (68.6, 261.2) | 87.63 (50.7, 171.4) | 0.006* | 137.5 (68.2,272.0) | 87.0 (50.4,143.8) | <0.001* |
| 24h TP, ms^2^ | 1263.0 (805.6, 2117.8) | 1289.3 (832.4, 2143.1) | 1213.09 (748.9, 2061.3) | 0.176 | 1269.8 (799.9,2143.1) | 1207.1 (744.8,1990.5) | 0.297 |
| LF/HF | 2.1 (1.3, 3.6) | 2.0 (1.3, 3.2) | 2.5 (1.5, 4.4) | 0.037* | 1.8 (1.2,2.7) | 2.8 (1.6,4.7) | <0.001* |
| 7-day hematoma volume, mL | 10.2 (5.3, 18.0) | 6.1 (4.2,10.2) | 17.1(12.0,21.3) | <0.001* | 8.0 (5.1,12.6) | 13.2 (7.2,19.4) | <0.001* |
| 7-day PHE, mL | 20.2 (10.0, 41.0) | 11.4 (7.5,16.4) | 44.1 (34.1,60.5) | <0.001* | 11.6 (7.1,19.2) | 39.2 (25.7,57.9) | <0.001* |
| rPHE | 2.0 (1.6, 2.8) | 1.7 (1.4,1.9) | 2.7 (2.4,3.3) | <0.001* | 1.6 (1.3,1.7) | 2.8 (2.4,3.3) | <0.001* |
| ICH score | 1.0 (0.0,1.0) | 0.0 (0.0, 1.0) | 1.0 (0.0,1.0) | 0.001* | 0.0 (0.0,1.0) | 1.0 (0.0,1.0) | 0.659 |
| mRS | 2.0 (1.0, 3.0) | 2.0 (1.0, 2.0) | 3.0 (3.0, 4.0) | <0.001* | 2.0 (1.0,3.0) | 3.0 (2.0,4.0) | <0.001* |

Note: BMI, body mass index; CHD, coronary heart disease; CCB, calcium channel blocker; CT, computed tomography; ACEI, angiotensin-converting enzyme inhibitor; ARB, angiotensin receptor blocker; SBP, systolic blood pressure; DBP, diastolic blood pressure; NIHSS, National Institutes of Health Stroke Scale; GCS, Glasgow Coma Scale; HV, hematoma volume; IVH, intraventricular hemorrhage; ECG, electrocardiogram; SDNN, standard deviation of NN intervals; RMSSD, root mean square of successive differences between adjacent NN intervals; NN50, number of adjacent NN intervals differing by >50 ms; pNN50, percentage of adjacent NN intervals differing by more than 50 ms; ULF, ultralow-frequency; VLF, very low-frequency; LF, low-frequency, HF, high-frequency; TP, total power; ICH, intracerebral hemorrhage; PHE, perihematomal edema; rPHE, relative perihematomal edema.

**Supplementary Table 2. Model performance for five machine learning algorithms and comparison with ICH score.**

|  | AUC | AP | Accuracy | Precision | Recall | F1 Score | P for Delong's test with ICH score |
| --- | --- | --- | --- | --- | --- | --- | --- |
| LR | 0.865(0.798 - 0.932) | 0.852(0.778 - 0.925) | 0.786(0.708 - 0.864) | 0.776(0.704 - 0.848) | 0.731(0.616 - 0.846) | 0.752(0.658 - 0.845) | <0.001 |
| SVC | 0.869(0.804 - 0.935) | 0.857(0.785 - 0.929) | 0.792(0.72 - 0.865) | 0.795(0.72 - 0.87) | 0.724(0.623 - 0.826) | 0.757(0.669 - 0.844) | <0.001 |
| RF | 0.883(0.835 - 0.93) | 0.852(0.782 - 0.922) | 0.811(0.762 - 0.86) | 0.83(0.763 - 0.898) | 0.737(0.669 - 0.806) | 0.778(0.721 - 0.836) | <0.001 |
| AdaBoost | 0.876(0.823 - 0.929) | 0.86(0.8 - 0.92) | 0.805(0.73 - 0.88) | 0.766(0.684 - 0.848) | 0.823(0.749 - 0.898) | 0.793(0.715 - 0.871) | <0.001 |
| XGBoost | 0.883(0.833 - 0.934) | 0.867(0.813 - 0.921) | 0.805(0.743 - 0.867) | 0.775(0.71 - 0.841) | 0.801(0.729 - 0.874) | 0.787(0.722 - 0.853) | <0.001 |

LR, Logistic Regression; SVM, Support Vector Machine; RF, Random Forest; AdaBoost, Adaptive Boosting; XGBoost, eXtreme Gradient Boosting; AUC, area under curve; ICH, intracerebral hemorrhage.

**Supplementary Table 3. Optimal hyperparameters for five machine learning algorithms**

|  | Hyperparameters |
| --- | --- |
| LR | C: 0.14843795619513322 |
| SVC | C: 0.01950674212801133 |
| RF | n_estimators: 58,  max_depth: 6,  max_features: 7,  min_samples_leaf: 0.047071160040383496,  min_samples_split: 0.01331878770097732 |
| AdaBoost | n_estimators: 73,  learning_rate: 0.11321766702498594,  algorithm: SAMME |
| XGBoost | n_estimators: 98,  learning_rate: 0.04348269525510873,  max_depth: 4,  gamma: 0.47954710929227745,  subsample: 0.506401218300208,  colsample_bytree: 0.4346203392759129,  lambda: 4.1937485304368085,  alpha: 4.26666738046644 |

LR, Logistic Regression; SVM, Support Vector Machine; RF, Random Forest; AdaBoost, Adaptive Boosting; XGBoost, eXtreme Gradient Boosting; AUC, area under curve; ICH, intracerebral hemorrhage.


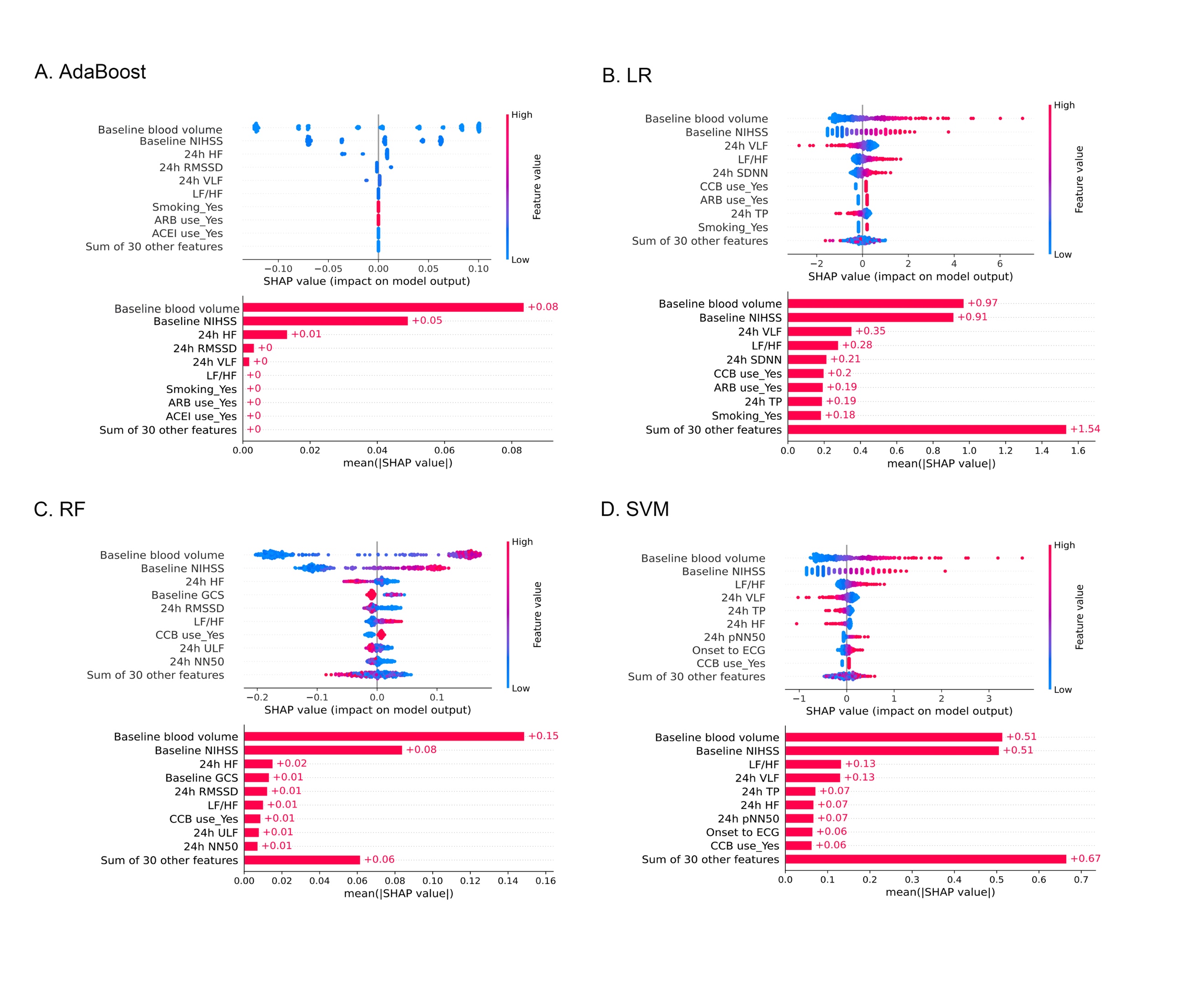


**Supplementary Figure 1. Other 4 machine learning models.**

LR, Logistic Regression; SVM, Support Vector Machine; RF, Random Forest; AdaBoost, Adaptive Boosting; XGBoost, eXtreme Gradient Boosting; ICH, intracerebral hemorrhage; SHAP, SHapley Additive exPlanations; BMI, body mass index; CHD, coronary heart disease; CCB, calcium channel blocker, ACEI, angiotensin-converting enzyme inhibitor; ARB, angiotensin receptor blocker; SBP, systolic blood pressure; DBP, diastolic blood pressure; NIHSS, National Institute of Health stroke scale; GCS, Glasgow Coma Scale; IVH, intraventricular hemorrhage; ECG, electrocardiography; SDNN, standard deviation of NN intervals; RMSSD, the root mean square of the differences between adjacent NN intervals; NN50, adjacent NN intervals varying by more than 50 milliseconds; pNN50, percentage of adjacent NN intervals varying by more than 50 milliseconds; ULF, ultralow-frequency; VLF, very low-frequency; LF, low-frequency, HF, high-frequency; TP, total power; ICH, intracerebral hemorrhage.
